# The Asian Synthetic Cell Initiative: highlights from the first SynCell Asia workshop

**DOI:** 10.1093/nsr/nwae377

**Published:** 2024-10-28

**Authors:** Meifang Fu, Xuefei Li, Weijie Zhao

**Affiliations:** Shenzhen Institute of Synthetic Biology, Shenzhen Institutes of Advanced Technology, Chinese Academy of Sciences; Shenzhen Institute of Synthetic Biology, Shenzhen Institutes of Advanced Technology, Chinese Academy of Sciences; Shenzhen Institute of Synthetic Biology, Shenzhen Institutes of Advanced Technology, Chinese Academy of Sciences


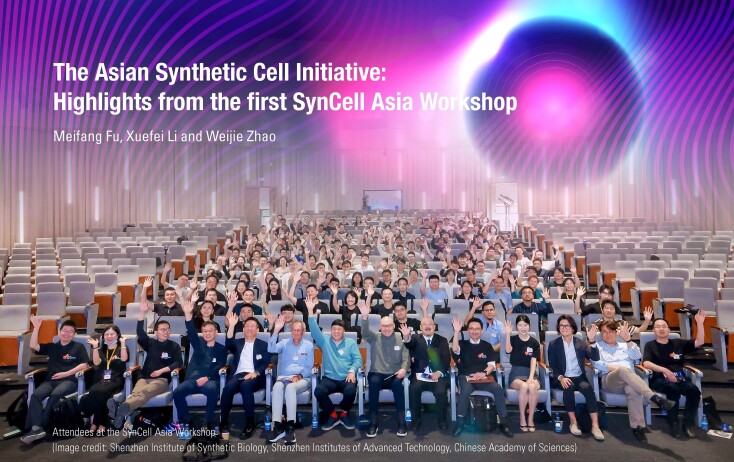


The complexity of life has captivated humanity for generations, driving the quest to understand the fundamentals of its origins. As early as the nineteenth century, scientists endeavored to artificially synthesize organic molecules from inorganic compounds. Over time, efforts have been made to glimpse into the components of life.

Over the past three decades, an increasing number of researchers have embraced the challenge of building a synthetic cell from scratch. However, this ambitious endeavor has often been fragmented, with studies focusing on one or two components of life. The building of an entire cell, however, necessitates multidisciplinary integration and holistic coordination.

Regional collaborations in synthetic cell research have been initiated in both Europe and the USA, leading to the formation of the European Synthetic Cell Initiative and the Build-a-Cell organization, respectively.

In October 2023, the Asian Synthetic Cell Initiative (SynCell Asia) was launched during the Asian Synthetic Biology Association (ASBA) Workshop on Biofoundry and Synthetic Cell. The first SynCell Asia workshop was subsequently held in April 2024 in Shenzhen, China. This event was co-hosted by the Shenzhen Institute of Synthetic Biology at the Shenzhen Institutes of Advanced Technology, Chinese Academy of Sciences (CAS) and ASBA. It brought together researchers from 25 institutions across Asia, including China, Japan, South Korea, Singapore, Thailand and Malaysia. During this workshop, a memorandum of understanding was signed, which officially launched the pan-Asia research collaboration on synthetic cell research.

The workshop featured four key sessions: SynCell Membrane and Replication, SynCell Transcription and Translation, SynCell Metabolism, and SynCell Technology and Application. Additionally, there was a Pan-Asian Strategic discussion, with each session followed by an in-depth panel discussion.

In this report, we aim to highlight the challenges and expectations of synthetic cell research that were presented during the workshop, with the hope of inspiring further discussion and cooperation.

## THE FIRST STEP TO SYNTHESIZE A CELL: MEMBRANE COMPARTMENTALIZATION AND REPLICATION

Membrane compartmentalization plays a pivotal role in the construction of synthetic cells, as it protects cellular metabolism and regulates the responses of the cells to the extracellular environment. In the first session of the workshop, ‘SynCell Membrane and Replication’, attendees discussed how to synthesize artificial cell membranes that are both stable and flexible, enabling the synthesized cells to maintain an integrated structure and be replicable simultaneously.

Liposomes are an excellent platform for integrating cellular functions with increasing complexity in the construction of synthetic cells. Several researchers presented their efforts to enhance the robustness and functions of liposomes.

Additionally, scientists are exploring non-liposome systems that may function as artificial organelles. For instance, the construction of artificial membraneless organelles within synthetic cells has been discussed. These organelles would be capable of the spatio-temporal control of intracellular biochemical reactions, responding to external signals and stimulating signal transduction.

Challenges remain in synthetic cell division. Currently, it is very difficult to divide a synthetic cell through biological methods, such as the reconstitution of division cytoskeletons. On the other hand, physical stimuli, such as osmotic shock, seem more feasible for achieving division. Continued efforts are needed in this field, with the belief that such division could be achieved in the next 5 years.

## BUILDING THE DNA–RNA–PROTEIN MACHINERY

One of the fundamental requirements of synthetic cells is the ability to synthesize their own proteins, necessitating the reconstitution of transcription and translation processes from the bottom up. In the second session of the workshop, ‘SynCell Transcription and Translation’, attendees discussed the challenges and future directions.

The ribosome—an organelle that is composed of RNAs and proteins—is responsible for translating messenger RNAs into proteins. The construction of artificial ribosomes is a critical step in building the DNA–RNA–protein machinery. However, this has not yet been accomplished due to the complex structure of the ribosome—it consists of 54 protein subunits and 3 rRNAs in E-coli—and the elusive understanding of the key assembly processes.

One famous piece of machinery that is required for protein translation is known as the PURE (Protein synthesis Using Recombinant Elements) system. Currently, the expression level of the PURE system is insufficient to produce all its components, meaning that the artificial translation system cannot sustain itself independently.

Despite these challenges, scientists remain optimistic. Attendees at the workshop anticipated that, with sufficient funding, a self-sustaining PURE system, or ‘PURE generating PURE’, could be achieved within the next 5–10 years.

## MAKE THE CELL ‘ALIVE’: SYNCELL METABOLISM

Metabolism is critical for providing the building blocks, energy and redox balance to support the self-regeneration of macromolecules. Efforts to design artificial metabolism for synthetic cells have focused on improving the efficiency of the enzymatic reactions, as well as enhancing metabolic regulation.

However, challenges remain. Metabolism processes are precisely regulated by various biomolecules, making it difficult to control the relative expression levels of enzymes and to sequentially and dynamically regulate the activity of metabolism cascades in synthetic cells. Additionally, the disparity between the physical conditions of living and artificial cells further complicates the design of SynCell metabolism.

Traditional approaches for metabolism pathway design focus on increasing protein productivity, while the design of an artificial metabolism for synthetic cells requires the generation of a coordinated and balanced metabolic system.

## APPLICATIONS OF SYNTHETIC CELLS

Attendees have extensively discussed the real-world impact of synthetic cell research, highlighting its applications in industry, scientific research and other fields.

For applications in industry, the opportunities lie in biomedicine and biomanufacturing, in which the identification of widely embraced ‘killer applications’ could promote collaboration. It is also suggested that these technologies should address the unique societal challenges that are faced by different countries.

For applications in scientific research, synthetic cells can serve as ideal research models for quantitative understanding, which is challenging in complex living systems. For example, synthetic cells can be used to study toxic enzymes under well-controlled conditions.

The ‘build to understand’ principle is a classic tenet of scientific research and SynCell approaches will undoubtedly advance our understanding of fundamental questions about life, and may help in exploring different forms of life that could exist on other planets. Connections between the synthetic cell and astrobiology communities have already emerged in Japan and other countries.

Synthetic cell research may also drive technological developments, similarly to the impact of the next-generation sequencing methods that resulted from the Human Genome Project. For instance, parameter search devices for high-throughput data acquisition under varying environmental conditions could potentially be developed.

## STANDARDIZATION FOR SYNCELL

Standardization is crucial in most scientific fields, and synthetic cell research is no exception. However, it may still be too early to define a universal synthetic cell system. As an attendee stated, ‘we are still at an exploratory phase and throwing pitches in many ways.’ Therefore, a careful balance must be struck between standardization and creativity, as premature standardization could hinder innovation in this rapidly developing field.

The key challenge to standardization is the lack of both data and the understanding of synthetic cells. However, for platform technologies such as cell-free protein expression, some form of standardization is ready to begin.

## BIOSAFETY CONCERNS

Attendees also discussed the potential biosafety dangers and regulatory policy of SynCell.

It is generally agreed that the risk is currently minimal, as we are still far from synthesizing fully self-replicating cells. The synthetic cells that are constructed from the bottom up are well controlled and more easily modified compared with other biotechnologies such as cell or virus engineering.

Instead of causing safety issues, synthetic cells could be used to study significant safety concerns, such as DNA transfer and natural evolution problems. It was also suggested that DNA transfer in synthetic cells could be used for drug delivery and other applications to address ongoing challenges in the biomedical field.

For biosafety regulation, it is suggested to respect pre-existing regulations. Synthetic cells could be regulated based on their product properties. For example, in the case that some toxic proteins have been produced, they can be regulated under existing frameworks for toxic compounds. A more complex case might involve the production of some unknown compounds, which could follow existing regulations for unknown compounds.

Regulations can also differ, depending on the application scenarios. If the purpose is to produce chemicals, existing regulations for the engineering of microbes can be followed, and synthetic cells might not pose a serious imminent threat in this context. However, special attention should be given to synthetic cells that are intended to be released into the environment.

## PAN-ASIAN STRATEGY FOR SYNCELL: DIVERSITY AND COOPERATION

During the Pan-Asian Strategic discussion, attendees deliberated on building SynCell communities through agenda coordination, funding, education and the formation of working groups.

The importance of funding support was emphasized. Currently, an Asian synthetic cell association might not secure sufficient funding for cross-continent projects independently. However, synthetic biology communities in Asia—especially in China, Korea and Japan—have received significant governmental support. These communities are ready to allocate some funding and resources for synthetic cell projects, which can be used to initiate flagship projects.

Countries that are open to GMOs (genetically modified organisms), such as Singapore, face no major hurdles in funding synthetic cells. However, there is a general concern that governments might pursue more immediate applications, making it challenging to secure long-term funding if synthetic cells are only for basic research.

Additionally, when applying for funding, careful consideration should be given to avoid the phrase ‘creating life’ to prevent unnecessary public sensitivity.

Global cooperation is extremely important for SynCell research, but must be approached carefully. As an idea-oriented (but not task-oriented) project, SynCell research encompasses diverse ideas, approaches and tools, and each country has different societies, budgets and cultures.

The SynCell project is interdisciplinary, involving biochemists, chemists, physicists and engineers. Attendees also suggested the inclusion of computer and social scientists, to enable broader discussions.

To encourage young researchers to enter this field, attendees proposed the organization of summer/winter schools and competitions for SynCell, similar to the International Genetically Engineered Machine (iGEM) competition for synthetic biology. In addition, exchange programs for students and researchers could naturally and smoothly promote communication between the countries, as young scientists are the main driving force of this project.

Other suggestions for promoting the SynCell initiative and workshops include: (i) focusing on different topics each year to attract scientists from various fields, which can also help in securing funding; (ii) establishing a global SynCell consortium to promote the community; (iii) maintaining continuous discussions, either in person or online; and (iv) standardizing synthetic cell concepts and experimental procedures through bio-foundries.

The first SynCell Asia workshop took place at a time at which global cooperation for synthetic cell research was on the rise. The workshop provided each task force with a better understanding of the strengths of the collaborative partners, laying the foundation for improved research activities within the research space.

## WORKSHOP ORGANIZERS AND INVITED GUESTS (ORDERED BY LAST NAME)

Matthew Wook Chang (National University of Singapore, Singapore), Byung-Kwan Cho (Korea Advanced Institute of Science & Technology, South Korea), Xixian Chen (Singapore Institute of Food and Biotechnology Innovation, Agency for Science, Technology and Research, Singapore), Zhuojun Dai (Shenzhen Institutes of Advanced Technology, CAS, China), Nannan Deng (Shanghai Jiaotong University, China), Julius Fredens (National University of Singapore, Singapore), Xiongfei Fu (Shenzhen Institutes of Advanced Technology, CAS, China), Jee Loon Foo (National Centre for Engineering Biology and National University of Singapore, Singapore), Xiaojun Han (Harbin Institute of Technology, China), ChunLoong Ho (Shenzhen Institutes of Advanced Technology, CAS, China), Norikazu Ichihashi (The University of Tokyo, Japan), Pattarawan Instasian (Vidyasirimedhi Institute of Science and Technology, Thailand), Fan Jin (Shenzhen Institutes of Advanced Technology, CAS, China), Daisuke Kiga (Waseda University, Japan), Dong-Myung Kim (Chungnam National University, South Korea), Akihiko Kondo (Kobe University, Japan), Jian Li (Shanghai Technology University, China), Junbai Li (Institute of Chemistry, CAS, China), Xuefei Li (Shenzhen Institutes of Advanced Technology, CAS, China), Chenli Liu (Shenzhen Institutes of Advanced Technology, CAS, China), Jianbo Liu (Hunan University, China), Chunbo Lou (Shenzhen Institutes of Advanced Technology, CAS, China), Yuan Lu (Tsinghua University, China), Wataru Mizunashi (New Energy and Industrial Technology Development Organization, Japan), Yan Qiao (Institute of Chemistry, CAS, China), Ahmad Bazli Ramzi (Universiti Kebangsaan Malaysia, Malaysia), Sang-Woo Seo (Seoul National University, South Korea), Yoshihiro Shimizu (RIKEN Center for Biosystems Dynamics Research, Japan), Kwanwoo Shin (Sogang University, South Korea), Longlong Si (Shenzhen Institutes of Advanced Technology, CAS, China), Tong Si (Shenzhen Institutes of Advanced Technology, CAS, China), Liangfei Tian (Zhejiang University, China), Duangthip Trisrivirat (Vidyasirimedhi Institute of Science and Technology, Thailand), Fei Yan (Shenzhen Institutes of Advanced Technology, CAS, China), Bo Zheng (Shenzhen Bay Laboratory, China) and Chao Zhong (Shenzhen Institutes of Advanced Technology, CAS, China).

## Notes

Authors: Meifang Fu and Xuefei Li are associate professors in Shenzhen Institute of Advanced Technology, CAS; Weijie Zhao is news and science editor of NSR.

Art Editor: Xiaoling Yu.

